# The data set describing cognitive performance after varenicline administration in a 3-choice serial reaction time task in rats

**DOI:** 10.1016/j.dib.2017.02.050

**Published:** 2017-03-03

**Authors:** Yu Ohmura, Hitomi Sasamori, Iku Tsutsui-Kimura, Takeshi Izumi, Takayuki Yoshida, Mitsuhiro Yoshioka

**Affiliations:** a Department of Neuropharmacology, Hokkaido University Graduate School of Medicine, Japan; b Hokkaido University School of Medicine, Japan; c Department of Neuropsychiatry, Keio University, School of Medicine, Tokyo, Japan; d Japan Society for the Promotion of Science, Japan

## Abstract

The data shows attentional function, impulsivity, motivation, motor function, and motor activity in rats treated with varenicline, a stop-smoking aid. The data also shows these parameters in rats treated with varenicline after acute/chronic nicotine administration. Our interpretation and discussion of these data were described in the article “Varenicline Provokes Impulsive Action by Stimulating α4β2 Nicotinic Acetylcholine Receptors in the Infralimbic Cortex in a Nicotine Exposure Status-Dependent Manner” (Ohmura et al., 2017) [1].

**Specifications Table**

**Table:** 

Subject area	*Biology, Psychology*
More specific subject area	*Psychopharmacology*
Type of data	*Excel file*
How data was acquired	*Aluminum operant chambers measuring 26 cm*×*26 cm*×*26 cm (Med Associates Inc., St. Albans, VT, USA) were used.*
Data format	*Raw*
Experimental factors	*Nicotine and/or varenicline administration*
Experimental features	*Adult male Wistar/ST rats were used.*
Data source location	*N/A*
Data accessibility	*All data were presented here.*

**Value of the data**

• The effects of stop-smoking aids on cognitive functions were of interest to many people.
• Some researchers might be interested in some parameters we did not focus on in the research paper [1].
• Clinicians can design human studies based on the animal data.
• The raw data would be useful if one would like to conduct reanalysis or meta-analysis.

## 1 Data

Data file 1 contains the raw data regarding Fig. 1 in [1]: several parameters (see below, Section 2) in a 3-choice serial reaction time task (3-CSRTT) in nicotine-naïve rats. Data file 2 contains the raw data regarding Fig. 2 in [1]: several parameters in the 3-CSRTT in nicotine-naïve rats after varenicline administration followed by microinjection of a α4β2 nicotinic acetylcholine receptor antagonist into the infralimbic cortex (IL). Data file 3 contains the raw data regarding Fig. 3 in [1]: several parameters in the 3-CSRTT in nicotine-naïve rats after acute s.c. injection of nicotine and varenicline. Data file 4 contains the raw data regarding Figs. 4 and 5 in [1]: several parameters in the 3-CSRTT after continuous infusion of nicotine and acute/repeated oral administration of varenicline.

## 2 Experimental design, materials and methods

We used a 3-choice serial reaction time task to assess impulsivity and other cognitive functions in rats [1]. We recorded seven behavioral parameters, as described below.

(a) Premature responses (counts per session): a measure of impulsivity.
(b) Accuracy (percentage of correct responses): a measure of attentional function.
(c) Omissions (counts per session): a measure of attentional function and motivation.
(d) Perseverative responses (counts per session): a measure of compulsivity.
(e) Responses during time-out (counts per session): a measure of motivation and motor activity.
(f) Correct response latency(s): a measure of attentional function, motivation, motor function, and decision time.
(g) Reward latency (s): a measure of motivation and motor function.

As for data file 1, nicotine naïve rats received acute s.c. injection of varenicline (0, 0.0075, 0.075, and 0.75 mg/kg) 60 min before the testing session.

As for data file 2, nicotine naïve rats received acute s.c. injection of varenicline (0 and 0.075 mg/kg) 60 min before the testing session and intra-IL microinjection of dihydro-β-erythroidine (DHβE; 0 and 6 μg/side), a preferential α4β2 nAChR antagonist, 10 min before the testing session.

As for data file 3, nicotine naïve rats received acute s.c. injection of varenicline (0, 0.0075, 0.075, and 0.75 mg/kg, s.c.) 60 min before the testing session and acute s.c. injection of nicotine (0 and 0.2 mg/kg) 10 min before the testing session. The dose of nicotine was the same as that used in our previous studies, demonstrating that nicotine induced impulsive action [2], [3].

As for data file 4, rats received acute oral administration of varenicline (0.075 mg/kg) 60 min before the testing session after 8 days of continuous nicotine (9 mg/kg/day, salt) or sodium tartrate administration with osmotic minipumps. The blood concentrations resulting from this dose in rats are comparable to those measured in heavy smokers [4], [5]. Repeated oral administration of varenicline (0.075 mg/kg, once per day) was conducted after stopping continuous nicotine infusion.
